# Minimum Lumen Area Indexed to Left Ventricular Mass to Identify Functionally Significant Left Main Coronary Stenoses

**DOI:** 10.1002/ccd.70026

**Published:** 2025-07-30

**Authors:** Giuseppe Patti, Leonardo Grisafi, Marco Mennuni, Luca Cumitini, Manuel Bosco, Domenico D'Amario, Martina Solli, Roberta Rosso, Matilde Villa, Gioel Gabrio Secco, Edoardo Elia, Giuseppe Colonna, Vincenzo Galiffa

**Affiliations:** ^1^ Department of Translational Medicine University of Eastern Piedmont Novara Italy; ^2^ Division of Cardiology Maggiore della Carità Hospital Novara Italy; ^3^ Santi Antonio e Biagio e Cesare Arrigo Hospital Alessandria Italy; ^4^ Vito Fazzi Hospital Lecce Italy

**Keywords:** angiographically intermediate, coronary artery disease, fractional flow reserve (FFR), intravascular ultrasound (IVUS), ROC analysis

## Abstract

**Background and Aim:**

Diagnosing significant left main coronary artery (LMCA) stenoses based on anatomical criteria remains challenging. The ARMYDA FINISH multicenter study evaluated whether indexing intravascular ultrasound (IVUS)‐derived minimum lumen area (MLA) provides greater diagnostic accuracy than unindexed MLA for detecting hemodynamically significant LMCA stenoses, defined as a fractional flow reserve (FFR) ≤ 0.80.

**Methods:**

Fifty‐two patients with angiographically‐intermediate isolated LMCA lesion were prospectively enrolled. All patients underwent IVUS and FFR measurements. Primary endpoint was the diagnostic accuracy of MLA indexed to height, body surface area (BSA), body mass index (BMI) and left ventricular (LV) echocardiographic mass in identifying hemodynamically significant LMCA stenoses compared to unindexed MLA. Secondary endpoints were the diagnostic sensitivity, specificity, and predictive values of indexed vs unindexed MLA cutoffs.

**Results:**

Overall, 40 patients (77%) had FFR  > 0.80 and 12 (23%) FFR  ≤ 0.80. MLA indexed to LV mass achieved the highest area under the receiver operating characteristic curve (AUC = 0.91, 95% CI: 0.83–0.99; *p* < 0.001), compared to unindexed MLA (AUC = 0.86) and MLA indexed to height, BSA or BMI (AUC range = 0.84–0.85). At a cutoff of 29 mm^2^/kg for MLA/LV mass, sensitivity and negative predictive value (NPV) were both 100%, and specificity was 70%. All MLA thresholds (whether indexed and unindexed) exhibited low positive predictive value (PPV), ranging from 46% to 55%.

**Conclusions:**

MLA/LV mass ratio may represent an accurate diagnostic tool for “ruling out” flow‐limiting LMCA lesions. Future large studies are needed to validate the MLA/LV mass ratio in diverse clinical settings and refine its cutoff values.

**Abbreviations**
AUC: area under the curve
EEM: external elastic membrane
FFR: fractional flow reserve
ICC: intra‐class correlation coefficient
IVSd: interventricular septal thickness in diastole
IVUS: intravascular ultrasound
LAD: left anterior descending (artery)
LCx: left circumflex (artery)
LMCA: left main coronary artery
LVEDD: left ventricular end‐diastolic diameter
LVEF: left ventricular ejection fraction
MLA: minimum lumen area
MLD: minimum luminal diameter
MMAR: myocardial mass at risk
NPV: negative predictive value
PPV: positive predictive value
PWd: posterior wall thickness in diastole
QCA: quantitative coronary angiography
ROC: receiver‐operating characteristic
RVD: reference vessel diameter

## 1 Introduction

An accurate evaluation of left main coronary artery (LMCA) stenoses is critical, due to the high morbidity and mortality associated with significant disease [1, 2]. The invasive coronary angiography alone showed insufficient accuracy in assessing the hemodynamic significance of intermediate LMCA lesions, misclassifying up to one‐third of patients [3, 4]. To address this limitation, the 2024 European Society of Cardiology (ESC) guidelines for chronic coronary syndromes recommended considering fractional flow reserve (FFR) for functional assessment (Class IIa, Level A) and intravascular ultrasound (IVUS) for anatomical evaluation (Class IIa, Level B) of angiographically intermediate LMCA stenoses [2]. In particular, an invasive FFR assessment was here effective for detecting flow‐limiting lesions and guiding the decision to defer or proceed with coronary revascularization based on values > 0.80 or ≤ 0.80, respectively [5].

Similarly, an accurate relationship between IVUS‐derived minimum lumen area (MLA) and functional significance of LMCA stenoses was established [6]. However, the optimal MLA cutoff for defining a significant LMCA lesion remains uncertain [4, 7, 8, 9, 10, 11]. Moreover, the use of fixed MLA thresholds was often associated with unsatisfactory sensitivity and negative predictive value (NPV), thus carrying the risk to refrain from revascularization in patients with functionally significant LMCA stenoses [9, 10]. From the above, it appears that the use of fixed MLA parameters could not be always accurate in detecting flow‐limiting vs non‐flow‐limiting LMCA stenoses, and therefore for this purpose “one MLA threshold does not fit all”. Thus, adjusting the MLA measures for anthropometric parameters or echocardiography‐derived left ventricular (LV) mass could ameliorate the correlation with hemodynamic significance of LMCA lesions. ARMYDA FINISH aimed at investigating whether, as compared with unindexed MLA cutoffs, the use of IVUS‐derived MLA adjusted for anthropometric parameters or LV mass improves the diagnostic accuracy in identifying the hemodynamic significance of LMCA stenoses.

## 2 Methods

This is a prospective, cohort study performed at three Italian institutions: “Maggiore della Carità Hospital” in Novara, “SS. Antonio e Biagio e Cesare Arrigo Hospital” in Alessandria, and “Vito Fazzi Hospital” in Lecce. The institutional review boards of the participating centers approved the protocol. Written informed consent was obtained from all subjects. Consecutive patients with the following inclusion criteria were considered: age ≥ 18 years; intermediate (25%–69%) LMCA stenosis by quantitative coronary angiography (QCA); angiographic disease involvement of the left anterior descending artery (LAD) or left circumflex (LCx) ostium (≤ 5 mm). Exclusion criteria were: angiographic ulceration, dissection, or thrombus in the LMCA; significant LAD or LCx disease (> 50% narrowing by QCA) or chronic total occlusion of the right coronary artery; prior myocardial infarction with wall motion abnormalities in LAD or LCx territories; previous coronary artery bypass grafting or previous percutaneous coronary intervention on LMCA; severe valvular heart disease; left ventricular ejection fraction (LVEF) ≤ 30%. Patients meeting the enrollment criteria were included as study population, where individual demographic, clinical, laboratory, echocardiographic and angiographic variables, as well as concomitant medical therapies, were collected. For the purpose of the study, the following other data were obtained in all participants:

− LV mass at trans‐thoracic echocardiography: it was obtained using the American Society of Echocardiography (ASE)/European Association of Cardiovascular Imaging (EACVI)‐recommended formula for the estimation of LV mass from LV linear dimensions: LV mass (g) = 0.8 × (1.04 × [(LVEDD + IVSd + PWd)3 − (LVEDD)3]) + 0.6, where LVEDD = LV end‐diastolic diameter, IVSd = interventricular septal thickness in diastole and PWd = posterior wall thickness in diastole [12].
− QCA data, in at least 3 LMCA views: the presence of an intermediate LMCA stenosis (25%–69%), LMCA minimum luminal diameter (MLD), reference vessel diameter (RVD), and diameter stenosis (%) was obtained through automated edge‐detection software (Philips).
− FFR measurement: all patients underwent physiological evaluation of intermediate LMCA stenosis using FFR. FFR assessment from the LMCA to the LAD was mandatory in all cases. Additional FFR measurement from the LMCA to the LCx was performed only if the vessel had a reference diameter ≥ 2.5 mm, as assessed by QCA, and was judged to supply a significant myocardial territory. All procedures followed a standardized protocol. Intracoronary nitroglycerin (300 µg) was administered to minimize vasospasm. A 0.014‐inch pressure guidewire (Philips OmniWire) was zeroed and then equalized at the tip of the guiding catheter (at least 5–10 beats of normalization) and then advanced at least three vessel diameters distal to the lesion beyond the stenosis first into the LAD and, when appropriate, into the LCx. Hyperemia was induced by intracoronary bolus administration of adenosine (200 µg) followed by a saline flush. In patients with ostial LMCA disease, the guiding catheter was carefully disengaged from the LMCA ostium after adenosine injection to avoid damping and ensure accurate aortic pressure recording. FFR was measured using a 2‐beat Pd/Pa average window, after at least 3–5 stable beats in sinus rhythm or 5–10 beats in atrial fibrillation, and at least two measurements were obtained per vessel. After each distal measurement was obtained, the wire was pulled back under continued hyperemia with continuous recording over a 20‐s period back to the ostium of the guiding catheter. A normalization check for drift was performed and documented. If a drift was evident (Pd/Pa measured at the level of the catheter tip < 0.98 or > 1.02), measurements were repeated. An FFR value ≤ 0.80 defined hemodynamically significant LMCA lesions. All procedural and hemodynamic data were digitally recorded and stored for offline analysis (Philips).
− IVUS parameters: after functional assessment, IVUS imaging was performed using a rotating 40‐MHz transducer within a 3.2‐F imaging sheath (Philips, Volcano), pulling back the ultrasound probe from LAD. The LMCA MLA was defined as the smallest luminal cross‐sectional area from the aorto‐ostium to the carina; frames located distal to the carina (including true ostial LAD/LCx) were excluded from the analysis. In the case of unsatisfactory visualization of LMCA ostium by IVUS from LAD, an IVUS imaging from LCx was also obtained, with the smaller MLA measurement being used. Two independent, experienced operators, blinded to FFR results, identified the MLA and external elastic membrane (EEM) area. Plaque burden at the MLA site was calculated as follows: (EEM area−MLA)/EEM area × 100 (%). Inter‐ and intra‐observer variability were assessed using the 2‐way random single measure intra‐class correlation coefficient (ICC) and the 1‐way random two‐measure ICC, respectively. Offline analysis was performed using dedicated software integrated into the Philips Volcano workstation (Philips, San Diego, CA, USA).
− Indexed MLA parameters: the following indexed parameters were considered:
  ◦ MLA/LV mass: mm^2^/kg
  ◦ MLA/Height: mm^2^/m
  ◦ MLA/body surface area (BSA): mm^2^/m^2^
  ◦ MLA/body mass index (BMI): mm^2^/(kg/m^2^)

In accordance with the study protocol, IVUS and FFR assessments were performed only after any significant distal coronary artery disease had been corrected. Adequate correction was confirmed by the absence of suboptimal stent expansion or residual stenosis at IVUS analysis and by the lack of a focal pressure step‐up at repeat hyperaemic pull‐back.

### 2.1 Study Endpoints

Primary endpoint: diagnostic accuracy of MLA indexed to LV mass, height, BSA, or BMI vs unindexed MLA in predicting flow‐limiting LMCA stenoses (FFR ≤ 0.80).

Secondary endpoint: to compare sensitivity, specificity, positive predictive value (PPV), and NPV of indexed MLA thresholds versus unindexed MLA cutoff in predicting flow‐limiting LMCA stenoses.

### 2.2 Statistical Analysis

As this was an exploratory, pilot observational study, no formal sample size calculation was performed. Categorical variables are presented as absolute numbers (percentages) and compared by the Fisher exact test, if the expected frequency was < 5, otherwise the *χ* ^2^ test was used. Continuous variables are expressed as mean ± standard deviation (SD) or median [interquartile range (IQR)], based on the distribution (normal vs. non‐normal) assessed via the Shapiro–Wilk test. Differences between continuous data were evaluated using the Student *t*‐test for normally distributed variables and Mann–Whitney *U*‐test for non‐normally distributed variables. Receiver operating characteristic (ROC) curves were generated to assess each metric's ability to identify lesions with FFR  ≤ 0.80; the Youden index was used to determine optimal thresholds. Variables were subsequently dichotomized based on the optimal cutoffs for indexed and unindexed MLA, and sensitivity, specificity, PPV and NPV were calculated. Sensitivity analyses were performed using ROC curves to evaluate whether diagnostic accuracy varied according to potential confounders. All *p* values were two‐sided, and a threshold < 0.05 was considered statistically significant. Statistical analyses were performed using STATA version 18.0 (StataCorp, College Station, TX).

## 3 Results

**Figure 1:**
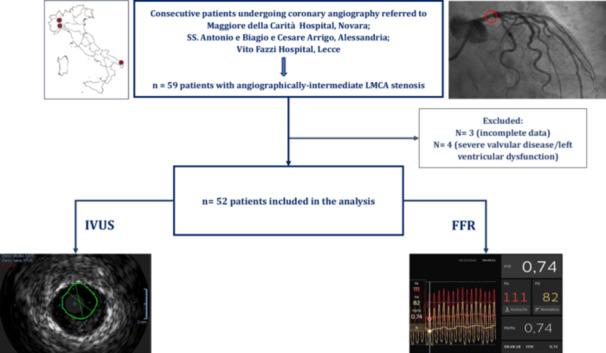
Study population. Overall, 59 consecutive patients initially met the inclusion criteria. After excluding 7 patients for incomplete data or valvular heart disease/left ventricular dysfunction, a total of 52 patients were ultimately included and underwent IVUS and FFR evaluation. FFR = fractional flow reserve, IVUS = intravascular ultrasound, LMCA = left main coronary artery. [Color figure can be viewed at wileyonlinelibrary.com]

Between May 2021 and October 2024, 59 consecutive Caucasian patients initially met the inclusion criteria. After excluding 7 patients for incomplete data or valvular heart disease/LV dysfunction, a total of 52 patients were ultimately included (Figure 1). Table 1 summarizes characteristics of the study population. A total of 40 patients (77%) exhibited FFR > 0.80 and 12 patients (23%) FFR ≤ 0.80. Patients with FFR > 0.80 and FFR ≤ 0.80 were similar for age, gender distribution and physical measurements, including height, weight, BMI, and BSA. Comorbidities and laboratory parameters were also comparable, as well as medical therapy, except for a higher prevalence of chronic use of calcium‐channel blockers (CCB) in the FFR ≤ 0.80 group (42% vs. 10% in the FFR > 0.80 group; *p* = 0.011). Echocardiographic findings are presented in Table 2. LVEF was overall preserved (56% [IQR: 49%–61%]), without difference between patients with FFR > 0.80 and ≤ 0.80. Median LV mass was numerically lower in the FFR > 0.80 group (187 vs. 218 g in the FFR ≤ 0.80 group; *p* = 0.17). Left atrial volume, IVSd thickness, and PWd thickness were similar in patients with FFR > 0.80 and ≤ 0.80. No patient with FFR > 0.80 underwent coronary revascularization, whereas all patients with FFR ≤ 0.80 received surgical (8.3%) or percutaneous (91.7%) revascularization.

**Table 1:** Characteristics of the study population.

	Overall *n* = 52	FFR > 0.80 *n* = 40 (77%)	FFR ≤ 0.80 *n* = 12 (23%)	*p* value
*Demographic and anthropometric characteristics*				
Age (years)	69 [65–78]	69 [65–79]	72 [62–77]	0.78
Male gender	40 (76.9)	31 (77.5)	9 (75.0)	0.86
Height (cm)	170 ± 9	170 ± 9	169 ± 7	0.72
BMI (kg/m^2^)	25 [24–29]	26 [23–29]	25 [24–27]	1.00
BSA (m^2^)	1.9 ± 0.2	1.9 ± 0.2	1.9 ± 0.2	0.93
*Cardiovascular risk factors and comorbidities*				
Systemic hypertension	36 (69.2)	27 (67.5)	9 (75.0)	0.62
Dyslipidemia	34 (65.4)	27 (67.5)	7 (58.3)	0.56
CKD	7 (13.5)	5 (12.5)	2 (16.7)	0.71
Diabetes	12 (23.1)	10 (25.0)	2 (16.7)	0.55
Smoking	30 (57.7)	25 (62.5)	5 (41.7)	0.20
AF	5 (9.6)	4 (10.0)	1 (8.3)	0.86
Previous PCI	19 (36.5)	17 (42.5)	2 (16.7)	0.10
*Chronic medical therapy*				
ACEi/ARBs	28 (53.8)	20 (50.0)	8 (66.7)	0.47
Beta‐blockers	39 (75.0)	30 (75.0)	9 (75.0)	1.00
CCBs	9 (17.3)	4 (10.0)	5 (41.7)	**0.011**
Nitrates	7 (13.5)	7 (17.5)	—	0.12
ASA	34 (65.4)	28 (70.0)	6 (50.0)	0.20
P2Y12 inhibitor	23 (44)	16 (40.0)	7 (58.3)	0.26
Statins	30 (57.7)	24 (60.0)	6 (50.0)	0.54
*Laboratory parameters*				
Hb (g/dL)	13.9 ± 1.8	13.9 ± 1.9	14.1 ± 1.5	0.68
eGFR (mL/min/1.73 m^2^)	70 [51–90]	65 [51–89]	78 [50–93]	0.72
LDL‐C (mg/dL)	86 ± 37	81 ± 36	103 ± 35	0.07
HbA1c (%)	5.8 [5.4–6.0]	5.9 [5.7–6.0]	5.7 [5.3–6.1]	0.57

*Note:* Data are expressed as absolute numbers (percentages) for categorical variables and mean ± standard deviation for continuous variables with normal distribution or median [interquartile range] for continuous variables with non‐normal distribution.

Abbreviations: ACEi/ARBs = angiotensin‐converting enzyme inhibitors/angiotensin receptor blockers, AF = atrial fibrillation, ASA = acetylsalicylic acid, BMI = body mass index, BSA = body surface area, CKD = chronic kidney disease, eGFR = estimated glomerular filtration rate, FFR = fractional flow reserve, Hb = haemoglobin, LDL‐C = low‐density lipoprotein cholesterol, PCI = percutaneous coronary intervention.

**Table 2:** Echocardiographic parameters.

	Overall *n* = 52	FFR > 0.80 *n* = 40 (77%)	FFR ≤ 0.80 *n* = 12 (23%)	*p* value
LVEF (%)	56.0 [49.0–61.0]	55.0 [48.0–61.0]	59.0 [55.0–61.5]	0.28
IVS thickness (mm)	11.3 ± 1.9	11.2 ± 1.9	11.8 ± 1.8	0.35
PW thickness (mm)	9.8 ± 1.9	9.7 ± 2.0	10.3 ± 1.6	0.30
EDD (mm)	48.0 [44.0–53.5]	48.0 [43.5–52.5]	49.5 [45.0–56.5]	0.57
EDS (mm)	34.3 ± 8.2	34.3 ± 7.8	34.2 ± 9.8	0.96
LV mass (g)	187.4 [153.1–239.9]	186.6 [149.4–230.3]	218.0 [158.6–291.0]	0.17
LV mass index (g/m^2^)	102.0 [84.8–122.0]	98.2 [82.6–115.7]	122.0 [89.4–143.2]	0.12
TAPSE (mm)	22.6 ± 4.7	22.6 ± 4.1	22.5 ± 6.6	0.94

*Note:* Data are expressed as mean ± standard deviation for continuous variables with normal distribution or median [interquartile range] for continuous variables with non‐normal distribution.

Abbreviations: EDD = end‐diastolic diameter, EDS = end‐systolic diameter, IVS = interventricular septum, LV = left ventricular, LVEF = left ventricular ejection fraction, PW = posterior wall, TAPSE = tricuspid annular plane systolic excursion.

Table 3 summarizes QCA and IVUS results. Right coronary artery dominance was highly prevalent, being observed in 92% of patients regardless of FFR status. In patients with FFR ≤ 0.80, the LMCA lesion was more frequently located at the bifurcation. In bifurcation lesions, the most common Medina classifications were 1,1,1 and 1,1,0 (45.8% and 37.5%, respectively). As expected, QCA analysis revealed that patients with FFR ≤ 0.80 exhibited a greater diameter stenosis (56.17 ± 6.31% vs. 43.73 ± 8.12% in those with FFR  > 0.80; *p* < 0.001), a smaller MLD (2.04 ± 0.66 vs. 2.54 ± 0.71 mm; *p* = 0.035) and longer lesion length (12.97 ± 6.36 mm vs. 8.47 ± 4.25 mm; *p* = 0.006). RVD at LMCA site was overall 5.05 ± 0.91 mm, similar in patients with FFR ≤ 0.80 and > 0.80. IVUS measurements revealed in the FFR ≤ 0.80 group a smaller MLA (4.40 [3.07–4.85] vs. 6.80 [5.10–9.75] mm^2^ in the FFR > 0.80 group; *p* < 0.001) and a greater plaque burden (72.78 ± 9.22 vs. 61.46  ± 12.05%; *p* = 0.004). The EEM area did not differ between the two groups (19.11 ± 4.13 vs. 20.25 ± 5.58 mm^2^; *p* = 0.52). Indexed MLA measures are reported in Table 4. Of note, the assessment of IVUS‐derived MLA demonstrated a high agreement degree, showing an interobserver ICC of 0.90 (95% CI: 0.84–0.94) and an intraobserver ICC of 0.92 (95% CI: 0.88–0.96). As compared with the FFR > 0.80 group, patients with FFR ≤ 0.80 had lower median MLA indexed to LV mass (20.50 vs. 37.85 mm^2^/kg; *p* < 0.001), MLA indexed to height (2.64 vs. 3.94 mm^2^/m; *p* < 0.001), MLA indexed to BSA (2.42 vs. 3.74 mm^2^/m^2^; *p* < 0.001), and MLA indexed to BMI [0.18 vs. 0.27 mm^2^/(kg/m^2^); *p* < 0.001].

**Table 3:** Procedural data.

	**Overall *n*** = **52**	**FFR** > **0.80 *n*** = **40 (77%)**	**FFR** ≤ **0.80 *n*** = **12 (23%)**	***p* value**
FFR	0.85 ± 0.08	0.89 ± 0.05	0.74 ± 0.05	**< 0.001**
*Coronary angiographic parameters*				
Right coronary dominance	48 (92.3)	37 (92.5)	11 (91.7)	0.92
LMCA lesion site				**0.012**
Ostium	19 (36.5)	18 (45.0)	1 (8.3)	
Shaft	9 (17.3)	8 (20.0)	1 (8.3)	
Bifurcation	24 (46.2)	14 (35.0)	10 (83.3)	
*Medina classification*				0.97
Medina 1,0,0	2 (8.3)	1 (7.1%)	1 (10.0%)	
Medina 1,1,0	9 (37.5)	5 (35.7%)	4 (40.0%)	
Medina 1,0,1	2 (8.3)	1 (7.1%)	1 (10.0%)	
Medina 1,1,1	11 (45.8)	7 (50.0%)	4 (40.0%)	
LMCA diameter stenosis (%)	46.60 ± 9.33	43.73 ± 8.12	56.17 ± 6.31	**< 0.001**
LMCA MLD (mm)	2.43 ± 0.73	2.54 ± 0.71	2.04 ± 0.66	**0.035**
LMCA RVD (mm)	5.05 ± 0.91	5.00 ± 0.98	5.22 ± 0.58	0.46
Lesion length	9.51 ± 5.12	8.47 ± 4.25	12.97 ± 6.36	**0.006**
*IVUS parameters*				
MLA (mm^2^)	5.85 [4.65–9.15]	6.80 [5.10–9.75]	4.40 [3.07–4.85]	**< 0.001**
EEM area (mm^2^)	19.98 ± 5.26	20.25 ± 5.58	19.11 ± 4.13	0.52
Plaque burden (%)	64.07 ± 12.35	61.46 ± 12.05	72.78 ± 9.22	**0.004**

*Note:* Data are expressed as absolute numbers (percentages) for categorical variables and mean ± standard deviation for continuous variables with normal distribution or median [interquartile range] for continuous variables with non‐normal distribution.

Abbreviations: EEM = external elastic membrane, FFR = fractional flow reserve, IVUS = intravascular ultrasound, LMCA = left main coronary artery, MLA = minimum lumen area, MLD = minimum lumen diameter, RVD = reference vessel diameter.

**Table 4:** Indexed MLA measures.

	Overall *n* = 52	FFR > 0.80 *n* = 40 (77%)	FFR ≤ 0.80 *n* = 12 (23%)	*p* value
MLA/LV Mass (mm^2^/Kg)	30.31 [23.43–43.06]	37.85 [27.05–51.38]	20.50 [16.77–25.21]	**< 0.001**
MLA/Height (mm^2^/m)	3.51 [2.68–5.36]	3.94 [3.11–5.67]	2.64 [1.72–2.90]	**< 0.001**
MLA/BSA (mm^2^/m^2^)	3.09 [2.47–4.63]	3.74 [2.87–4.91]	2.42 [1.54–2.78]	**< 0.001**
MLA/BMI [mm^2^/(Kg/m^2^)]	0.23 [0.18–0.31]	0.27 [0.20–0.36]	0.18 [0.12–0.19]	**< 0.001**

*Note:* Data are expressed as median [interquartile range].

Abbreviations: BMI = body mass index, BSA = body surface area, FFR = fractional flow reserve, LV = left ventricular, MLA = minimum luminal area.

### 3.1 ROC Analysis and Threshold Comparisons

**Figure 2:**
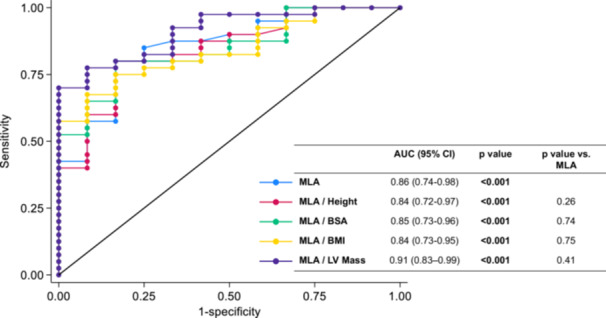
ROC curve analysis indicating the performance of indexed and unindexed MLA measures on the prediction of functionally significant LMCA stenosis (FFR ≤ 0.80). MLA/LV mass yielded the highest AUC at 0.91 (95% CI: 0.83–0.99; *p* < 0.001), outperforming unindexed MLA (AUC = 0.86). AUCs for MLA indexed to height, MLA indexed to BSA and MLA indexed to BMI were lower (0.84, 0.85, and 0.84, respectively). However, the differences in AUC between indexed MLA measures and unindexed MLA did not reach statistical significance (*p* values ranging from 0.26 to 0.75). AUC = area under the curve, BMI = body mass index, BSA = body surface area, FFR = fractional flow reserve, LMCA = left main coronary artery, LV = left ventricular, MLA = minimum lumen area, ROC = receiver‐operating characteristic. [Color figure can be viewed at wileyonlinelibrary.com]

Figure 2 depicts ROC curves for unindexed MLA and indexed MLA measures for the prediction of functionally significant LMCA stenosis (FFR ≤ 0.80) (primary study endpoint). MLA/LV mass yielded the highest area under the curve (AUC) at 0.91 (95% confidence interval [CI]: 0.83–0.99; *p* < 0.001), outperforming unindexed MLA (AUC = 0.86). AUCs for MLA indexed to height, MLA indexed to BSA and MLA indexed to BMI were lower (0.84, 0.85, and 0.84, respectively). AUC for plaque burden was the lowest [0.81 (0.67–0.95)]. However, the differences in AUC between indexed MLA measures and unindexed MLA did not reach statistical significance (*p* values ranging from 0.26 to 0.75).

**Figure 3:**
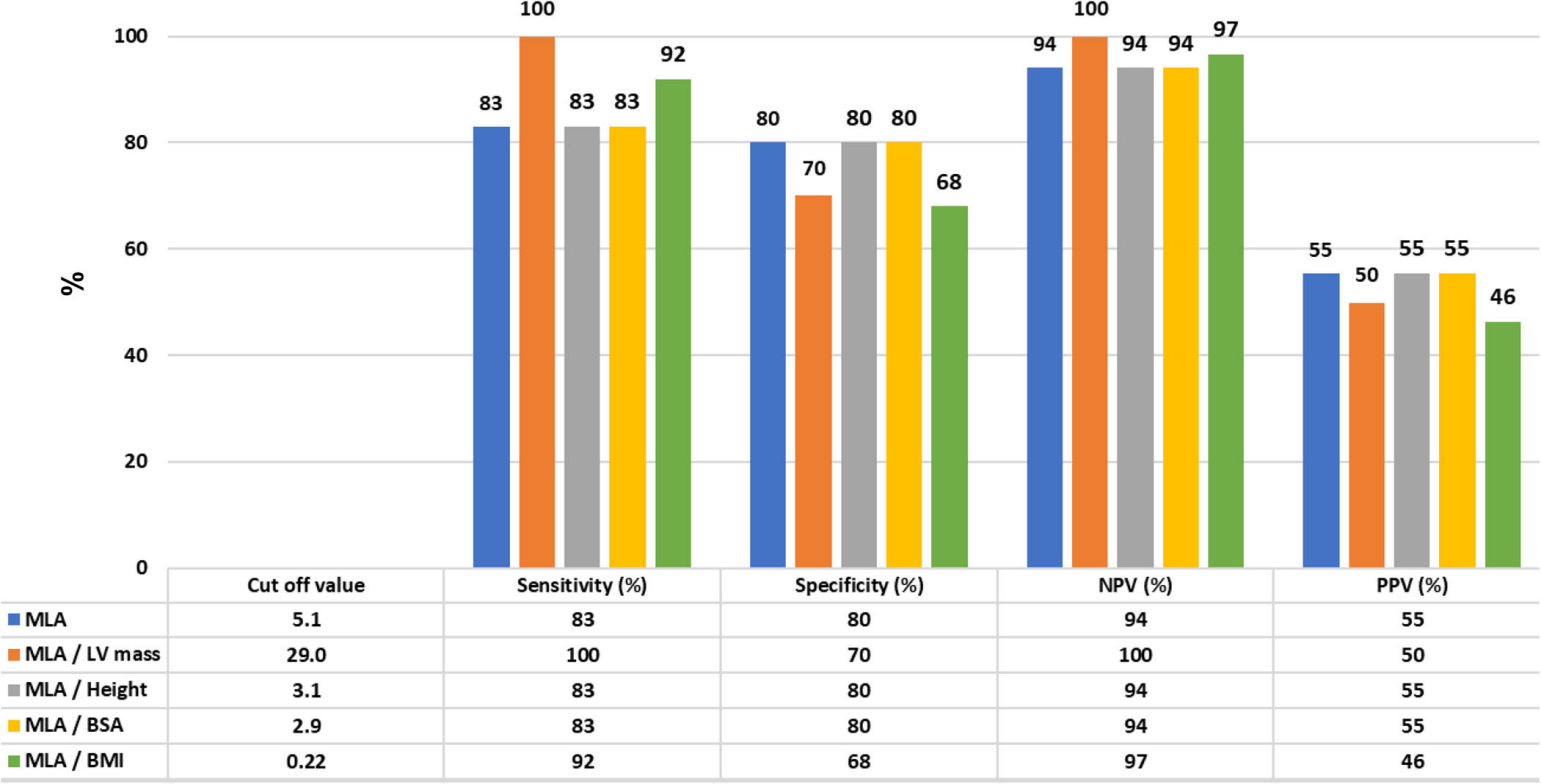
Overall performance of unindexed and indexed MLA cutoffs on the prediction of functionally significant LMCA stenosis (FFR ≤ 0.80). The unindexed MLA threshold of 5.1 mm^2^ provided a balanced 83% sensitivity and 80% specificity, with a 94% NPV and 55% PPV. The MLA/LV mass ratio at 29 mm^2^/kg had the highest sensitivity and NPV (both 100%), whereas specificity was 70%. MLA indexed to anthropometric measures (MLA/Height, MLA/BSA, or MLA/BMI) exhibited an intermediate performance. BMI = body mass index, BSA = body surface area, FFR = fractional flow reserve, LMCA = left main coronary artery, LV = left ventricular, MLA = minimum lumen area, NPV = negative predictive value, PPV = positive predictive value. [Color figure can be viewed at wileyonlinelibrary.com]

**Figure 4:**
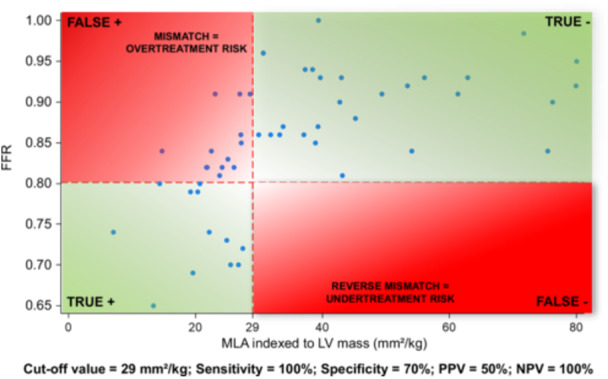
Individual data of MLA/LV mass ratio (mm²/Kg) and FFR values in the study population. The red dashed lines denote the following diagnostic cut‐off points: MLA/LV mass ratio of 29 mm^2^/Kg and FFR threshold of 0.80. Notably, no patient with MLA/LV mass ratio ≥ 29 mm^2^/kg had FFR ≤ 0.80, as visually indicated by the absence of data points in the bottom‐right quadrant of the plot; 50% of patients with MLA/LV mass ratio < 29 mm^2^/kg had FFR > 0.80. FFR = fractional flow reserve, LV = left ventricular, MLA = minimum lumen area, NPV = negative predictive value,PPV = positive predictive value. [Color figure can be viewed at wileyonlinelibrary.com]

**Figure 5:**
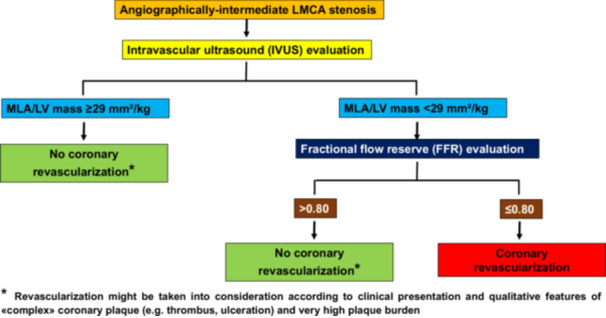
Proposed approach to angiographically‐intermediate LMCA stenoses. Patients initially undergo IVUS. If MLA/LV mass ratio results ≥ 29 mm^2^/kg, revascularization is not recommended*. If MLA/LV mass ratio is < 29 mm^2^/kg, FFR evaluation is mandatory. An FFR > 0.80 suggests no significant hemodynamic impact, and revascularization is not necessary*, while an FFR ≤ 0.80 indicates a flow‐limiting stenosis, warranting coronary revascularization. *Revascularization might be taken into consideration according to clinical presentation and qualitative features of «complex» coronary plaque (e.g., thrombus, ulceration) and very high plaque burden. FFR = fractional flow reserve, IVUS = intravascular ultrasound, LMCA = left main coronary artery, LV = left ventricular, MLA = minimum lumen area. [Color figure can be viewed at wileyonlinelibrary.com]

The following thresholds were then identified at AUC analysis: unindexed MLA of 5.1 mm^2^, MLA/LV mass of 29 mm^2^/kg, MLA/Height of 3.1 mm^2^/m, MLA/BSA of 2.9 mm^2^/m^2^ and MLA/BMI of 0.22 mm^2^/(kg/m^2^). Figure 3 illustrates sensitivity, specificity, PPV and NPV of these thresholds for predicting a functionally significant LMCA stenosis (FFR ≤ 0.80). The unindexed MLA threshold of 5.1 mm^2^ provided a balanced 83% sensitivity and 80% specificity, with a 94% NPV and 55% PPV. The MLA/LV mass ratio at 29 mm^2^/kg had the highest sensitivity and NPV (both 100%), whereas specificity was 70%. MLA indexed to anthropometric measures (MLA/Height, MLA/BSA or MLA/BMI) exhibited an intermediate performance. In particular, the scatterplot in Figure 4 shows individual data on the relationship between MLA/LV mass ratio (mm^2^/kg) and FFR values in the study population. Notably, no patient with MLA/LV mass ratio ≥ 29 mm^2^/kg had FFR ≤ 0.80, as visually indicated by the absence of data points in the bottom‐right quadrant of the plot; 50% of patients with MLA/LV mass ratio < 29 mm^2^/kg had FFR > 0.80. The scatterplot of individual data on the relationship between unindexed MLA (mm^2^) and FFR values in the study population is provided in Supporting Information S1: Figure 1. The sensitivity curve in Supporting Information S2: Figure 2 further illustrates that indexing MLA to LV mass achieved full sensitivity at a threshold of 29 mm^2^/kg. Supplementary Table 1 reports characteristics of patients stratified by the MLA/LV mass threshold of 29 mm^2^/kg. Out of the 52 subjects, 27 (52%) had MLA/LV mass ≥ 29 and 25 (48%) MLA/LV mass < 29 mm^2^/kg. These two groups showed no difference in age, sex distribution, basic anthropometric measures, cardiovascular risk factors, comorbidities and laboratory data. Patients with MLA/LV mass < 29 mm^2^/kg had a higher prevalence of nitrates and CCBs chronic treatment. IVSd thickness, PWd thickness and LV mass were also greater in the subgroup with MLA/LV mass < 29 mm^2^/kg. Supporting Information S3: Figure 3 illustrates a sensitivity ROC analysis showing that the chronic use of vasodilator agents (nitrates or CCBs) did not affect the predictive performance of the MLA/LV mass ratio for identifying FFR ≤ 0.80 (*p *= 0.72). The comparison of MLA/LV mass ratio performance for identifying FFR ≤ 0.80 according to FFR assessment strategy (Supporting Information S4: Figure 4) showed no statistically significant difference (*p* = 0.18) between LMCA‐to‐LAD only and LMCA‐to‐both LAD and LCx measurements. Moreover, there was no difference in the MLA/LV mass ratio performance for identifying FFR ≤ 0.80 according to LMCA lesion length < 8 mm or ≥ 8 mm (*p *= 0.76), as showed in Supporting Information S5: Figure 5.

## 4 Discussion

This study shows the potential advantage of adjusting IVUS‐derived MLA for patient‐specific variables over a fixed MLA measure to derive the functional significance of angiographically‐intermediate LMCA stenoses. In particular:

− As compared with unindexed MLA and MLA indexed to anthropometric measures (height, BSA and BMI), MLA indexed to echocardiographic LV mass had a greater discrimination power in detecting flow‐limiting LMCA lesions, defined as FFR ≤ 0.80.
− A cutoff of MLA/LV mass ratio ≥ 29 mm^2^/kg had an excellent “rule‐out” capacity, characterized by 100% sensitivity and NPV.
− All MLA‐based thresholds, whether indexed or unindexed, have modest specificity and PPV.

The applicability of IVUS‐derived anatomical plaque measures to represent functionally significant LMCA lesions and guide coronary revascularization derives from the greater discriminative power of MLA in LMCA lesions than in non‐LMCA stenoses [4, 8]. This was attributed to short lesion length and large vessel size in case of LMCA disease, as well as to the lack of side branches in ostial and shaft LMCA lesions. An MLA of 6.0 mm^2^ was traditionally considered to stratify flow‐limiting LMCA stenoses [4, 8]. This cutoff was primarily obtained from Murray law, extrapolating it from the ischemic threshold for MLA of lesions involving LAD or LCx arteries. However, the recent downgrading of such ischemic threshold for MLA of non‐LMCA stenoses [13, 14] suggests that, by applying the Murray law, the optimal MLA cutoff for representing flow‐limiting LMCA lesions might be < 6.0 mm^2^. This is corroborated by data indicating that a lower MLA threshold (4.5 mm^2^) prevents from overestimation of the functional significance of LMCA lesions, thereby decreasing the number of patients with mismatch (e.g., with anatomically severe, but functionally nonsevere, stenoses) and the percentage of unneeded operations/interventions [9]. Indeed, the latter evidence was obtained in Asian people, characterized by small body sizes and LMCA diameters, and it is well known that FFR results are influenced by reduced LMCA dimensions associated with lower BSA [11]. However, regardless of the MLA thresholds, the use of fixed, unindexed MLA measures was associated with reverse mismatch (e.g., patients with anatomically nonsevere, but functionally severe, lesions), leading to undertreatment [9]. Thus, we hypothesized that indexing the MLA of LMCA lesions to anthropometric or LV mass measures could decrease the occurrence of reverse mismatch. A previous investigation on Asian patients showed that, as compared with unindexed MLA measures, the adjustment of MLA for anthropometric parameters, such as BSA and BMI, did not improve the diagnostic accuracy in assessing the hemodynamic significance of LMCA stenoses [9]. ARMYDA‐FINISH, performed in a Caucasian population with overall a greater body size, showed similar results.

Interestingly, recent data have increasingly recognized the extent of myocardium subtended by the coronary lesion—also defined “myocardial mass at risk” (MMAR)—as a pivotal factor explaining the discrepancies between MLA and FFR measurements. In particular, an inverse correlation was demonstrated in intermediate coronary stenoses between amount of jeopardized myocardium and FFR values, suggesting that lesions supplying larger myocardial territories are more likely to exhibit a substantial FFR drop during hyperemia [15, 16]. Similarly, the extent of perfused myocardium independently contributed to the discrepancy between MLA severity and FFR results [17]. However, such studies had excluded patients with LMCA stenoses. In our work the adjustment of IVUS‐derived MLA for the echocardiographic LV mass in patients with angiographically‐intermediate LMCA stenoses was able to reflect both anatomical lesion severity and associated MMAR. In particular, an MLA/LV mass ratio ≥ 29 mm²/kg reliably excluded flow‐limiting stenoses, exhibiting 100% of sensitivity and NPV (vs. 83% and 94%, respectively, of unindexed MLA). Thus, indexing MLA to LV mass allowed to reduce the number of patients with reverse mismatch. Conversely, the low specificity and PPV indicate that a low MLA/LV mass ratio does not invariably reflect functionally significant LMCA disease, likely because of interindividual variations in myocardial demand, microvascular function, LV filling pressures and collateral circulation. To date, no previous study focusing on LMCA stenoses had specifically provided sensitivity, specificity, NPV and PPV for indexed MLA thresholds; therefore, our data fill a gap in the current literature.

In the Park's study, MLA was also adjusted for echocardiographic LV mass, but, differently from our investigation, this did not improve the diagnostic accuracy over unindexed MLA [9]. Comparing the two studies, it appears that the AUC for MLA/LV mass ratio was higher in the present work (0.91 vs. 0.83). These discrepancies in diagnostic accuracy and AUC measures might be explained by differences in baseline characteristics of the included populations, such as a higher LMCA reference diameter (mean 5.1 vs. 3.8 mm) and a greater LV mass (mean 201 vs. 163 g) in our cohort versus Park's cohort. Moreover, in the latter the LV mass in patients with FFR > 80 and FFR ≤ 0.80 was nearly identical, while we observed a lower LV mass in the FFR > 0.80 group (186.6 vs. 218 g in the FFR ≤ 0.80 group). Thus, the greater LMCA size and the higher variation of LV mass values in our population likely enhanced the discriminative power of the MLA/LV mass ratio. Of note, the modest specificity and PPV for all MLA‐based thresholds—whether indexed or unindexed—was consistent in the two investigations [9].

### 4.1 Limitations

Despite its prospective and multicenter design, ARMYDA‐FINISH should be considered in light of its limitations. First, the relatively small sample size and the imbalance between patients with FFR > 0.80 and ≤ 0.80 may limit the statistical power and generalizability of the findings. However, given the pilot nature of the study, the aim was to provide initial insights and hypothesis‐generating observations rather than definitive conclusions. Second, the reliance of QCA‐based definitions (25%–69% stenosis) may have affected the enrollment. Although FFR is a well‐validated tool, confounding factors, such as variations in LV end‐diastolic pressure or microvascular dysfunction, may influence the results of this technique; however, we attempted to mitigate such concern by excluding patients with wall motion abnormalities, severe LV dysfunction or valvular disease. Moreover, a potential limitation of the study is the vessel‐specific variability of FFR, particularly between the LAD and LCx. However, in our protocol, FFR in the LAD was mandatory in all patients, while LCx assessment was limited to vessels with a reference diameter ≥ 2.5 mm and large myocardial distribution. This approach was intended to standardize and optimize physiological evaluation of LM disease. Our cohort exhibited a very high (92%) prevalence of right coronary dominance, potentially limiting the applicability of the results to patients with balanced or left‐coronary dominance, where the MMAR for LMCA lesions is greater. Furthermore, we did not compare IVUS findings with other intravascular imaging modalities (e.g., optical coherence tomography) and did not evaluate subsequent clinical outcome. The inclusion of a Caucasian population may restrict the external validity, indicating the need for studies on more diverse cohorts. The absence of a centralized corelab for IVUS analysis also represents a limitation. However, to ensure measurement reliability, IVUS images were independently analyzed by two experienced operators who were blinded to the FFR results. Reproducibility was assessed and revealed high interobserver and intra‐observer ICC, supporting the robustness of the imaging data despite the lack of corelab adjudication. Another potential limitation is that LV mass estimation may be influenced by suboptimal echocardiographic image quality. However, to minimize this and improve generalizability, we used the ASE/EACVI‐recommended linear‐dimension method based on parasternal long‐axis views, which is less affected by suboptimal apical imaging and is widely applicable in routine clinical practice, even in centers without access to advanced imaging modalities [12]. Finally, our results apply to patients with isolated LMCA lesions; indeed, LMCA stenoses are often associated with disease in other coronary districts and by protocol we performed IVUS and FFR assessment after the IVUS and FFR‐guided correction of distal coronary artery disease, if present (Central Illustration 1).

**Central Illustration 1:**
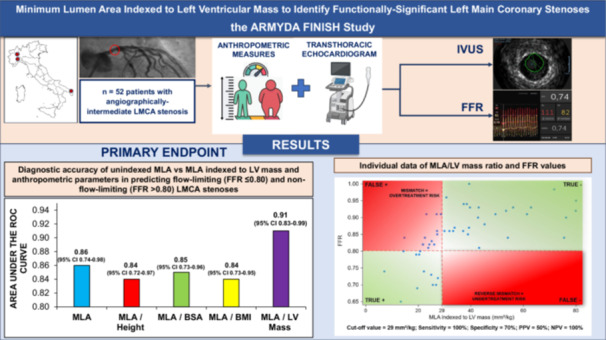
Study overview and main findings. In patients with angiographically‐intermediate LMCA disease, IVUS‐derived MLA indexed to echocardiographic LV mass achieved the highest area under the receiver operating characteristic curve (AUC = 0.91) in predicting flow‐limiting LMCA stenoses (FFR ≤ 0.80), compared to unindexed MLA (AUC = 0.86) and MLA indexed to height, BSA or BMI (AUC range = 0.84–0.85). At a cutoff of 29 mm^2^/kg for MLA/LV mass, sensitivity and NPV ranged both 100%, minimizing the risk of undertreatment. AUC = area under the curve, BMI = body mass index, BSA = body surface area, FFR = fractional flow reserve, IVUS = intravascular ultrasound, LMCA = left main coronary artery, LV = left ventricular, MLA = minimum lumen area, NPV = negative predictive value, PPV = positive predictive value, ROC = receiver‐operating characteristic. [Color figure can be viewed at wileyonlinelibrary.com]

## 5 Conclusions

In conclusion, ARMYDA‐FINISH indicates that indexing IVUS‐derived MLA to echocardiographic LV mass for LMCA lesions may represent a critical step toward a more precise clinical decision‐making process. In particular, the MLA/LV mass ratio can be considered an accurate screening tool for excluding flow‐limiting stenoses. Thus, the IVUS assessment, beside the qualitative evaluation of the LMCA plaque, here may obviate the need for FFR measurement when the MLA/LV mass ratio is ≥ 29 mm^2^/kg (Figure 5). This could translate into lower costs, while maintaining the appropriateness in the indication for coronary revascularization. Such MLA adjustment may be also useful in case of complex LMCA lesions, where FFR or noninvasive functional evaluation would be inaccurate, or in centers without FFR availability. In fact, the use of the MLA/LV mass ratio might reduce the occurrence of undertreatment and the number of patients in whom coronary revascularization is inappropriately omitted (e.g., a potential risk when the unindexed MLA threshold is utilized). Conversely, when the MLA/LV mass ratio is < 29 mm^2^/kg, the risk of overestimation of lesion significance and consequent overtreatment remains high; hence the FFR measurement in such case appears mandatory. However, future large‐scale studies are needed to validate the MLA/LV mass ratio in diverse clinical settings and refine its cutoff values.

## Ethics Statement

The institutional review boards of the participating centers approved the protocol.

## Consent

Written informed consent was obtained from all participants included in the study.

## Conflicts of Interest

G.P.: speaker/consultant fee from Amgen, Sanofi, Novartis, Daichi Sankyo, Amarin, Aurora BioPharma, Malesci, PIAM, Boheringer Ingheleim, Bayer, Pfizer/BMS, Astra Zeneca, Biotronik, Terumo, Medtronic, Abbott, Edwards, Amicus, Novo Nordisk, Chiesi. The other authors declare no conflicts of interest.

## Supporting information


Supplementary Figure 1.

Supplementary Figure 2.

Supplementary Figure 3.

Supplementary Figure 4.

Supplementary Figure 5.

**Supplementary Table 1:** Characteristics of the study population stratified by MLA/LV mass threshold (29 mm^2^/kg).

## Data Availability

Data supporting the findings of this study are available from the corresponding author upon reasonable request.
