# Supplementary material for: Minimum Lumen Area Indexed to Left Ventricular Mass to Identify Functionally Significant Left Main Coronary Stenoses
Source: Catheter Cardiovasc Interv. 2025 Jul 30;106(4):2207–17. doi: 10.1002/ccd.70026 (PMC12502031; doi:10.1002/ccd.70026)
Supplement: Supplementary file 1 — Supplementary Figure 1. [file CCD-106-2207-s004.pptx]

## Slide 1
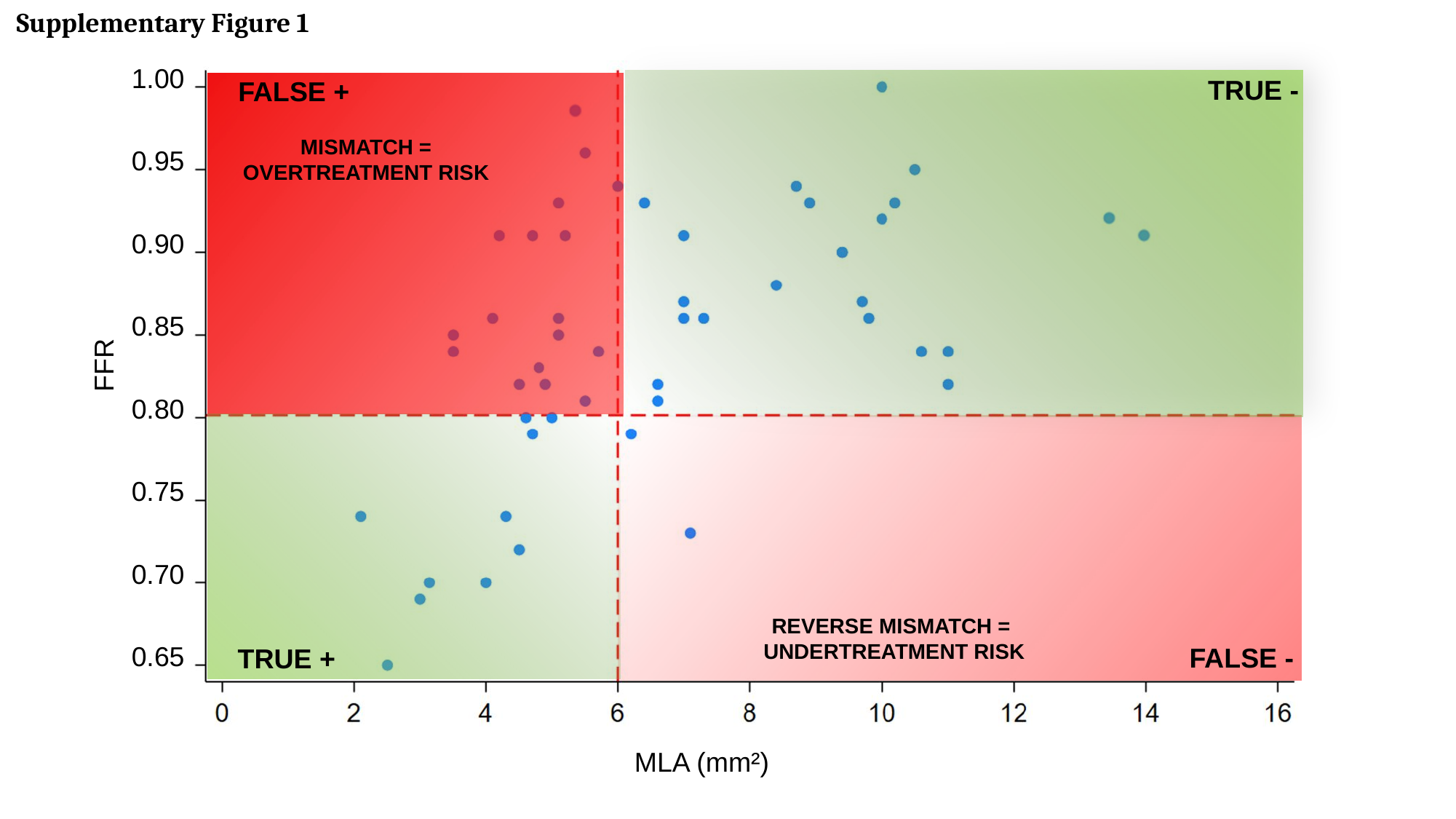

Supplementary Figure 1
| 1.00 |
| --- |
| 0.95 |
| 0.90 |
| 0.85 |
| 0.80 |
| 0.75 |
| 0.70 |
| 0.65 |
TRUE -
FALSE +
MISMATCH = OVERTREATMENT RISK
FFR
REVERSE MISMATCH =
UNDERTREATMENT RISK
FALSE -
TRUE +
MLA (mm²)
