# Supplementary figures and images for: Minimum Lumen Area Indexed to Left Ventricular Mass to Identify Functionally Significant Left Main Coronary Stenoses
Source: Catheter Cardiovasc Interv. 2025 Jul 30;106(4):2207–17. doi: 10.1002/ccd.70026 (PMC12502031; doi:10.1002/ccd.70026)

## Slide 1
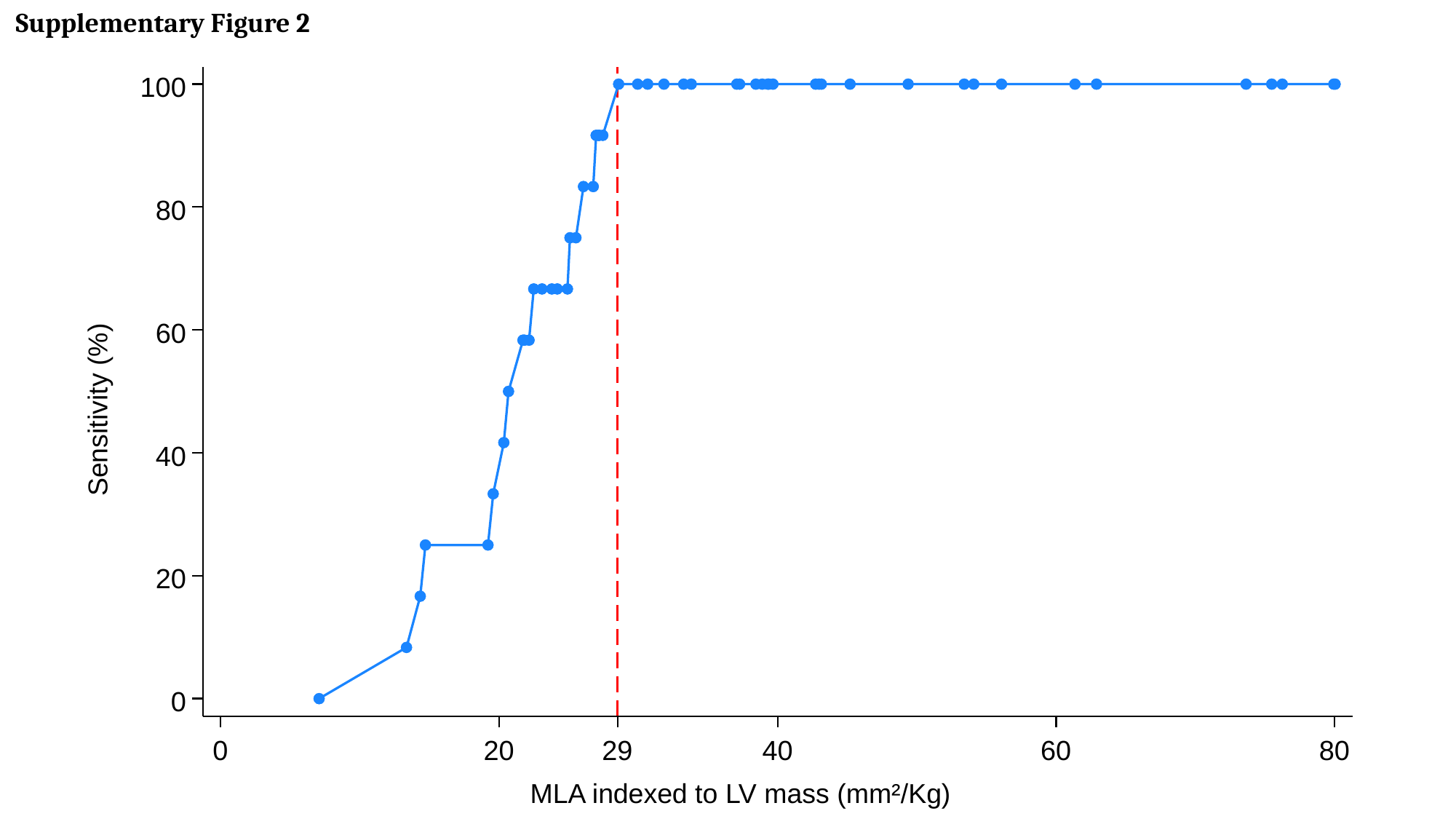

Supplementary Figure 2
Sensitivity (%)
MLA indexed to LV mass (mm²/Kg)

Supplement: Supplementary file 2 — Supplementary Figure 2. [file CCD-106-2207-s001.pptx]

## Slide 1
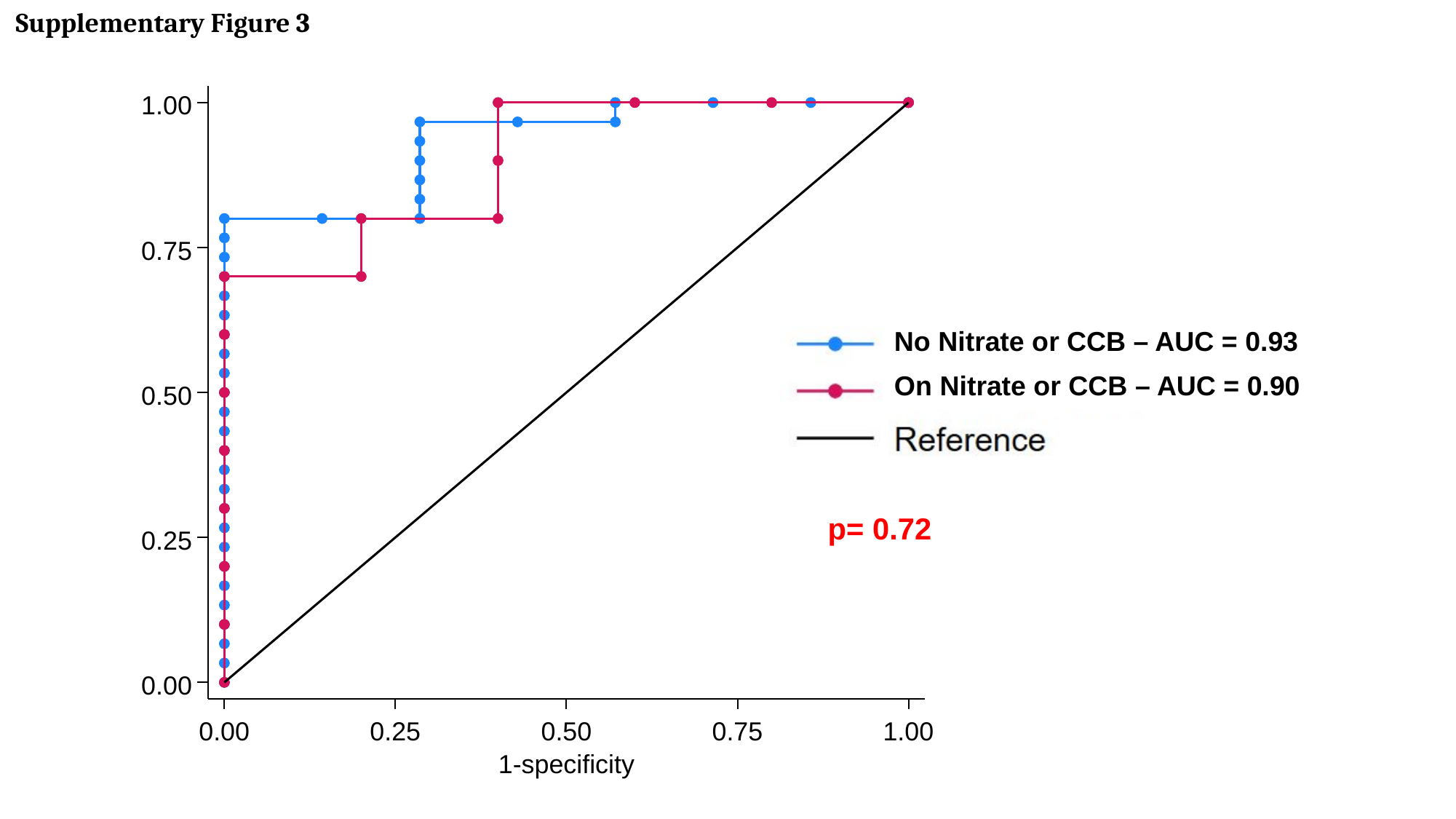

Supplementary Figure 3
| No Nitrate or CCB – AUC = 0.93 |
| --- |
| On Nitrate or CCB – AUC = 0.90 |
p= 0.72

Supplement: Supplementary file 3 — Supplementary Figure 3. [file CCD-106-2207-s003.pptx]

## Slide 1
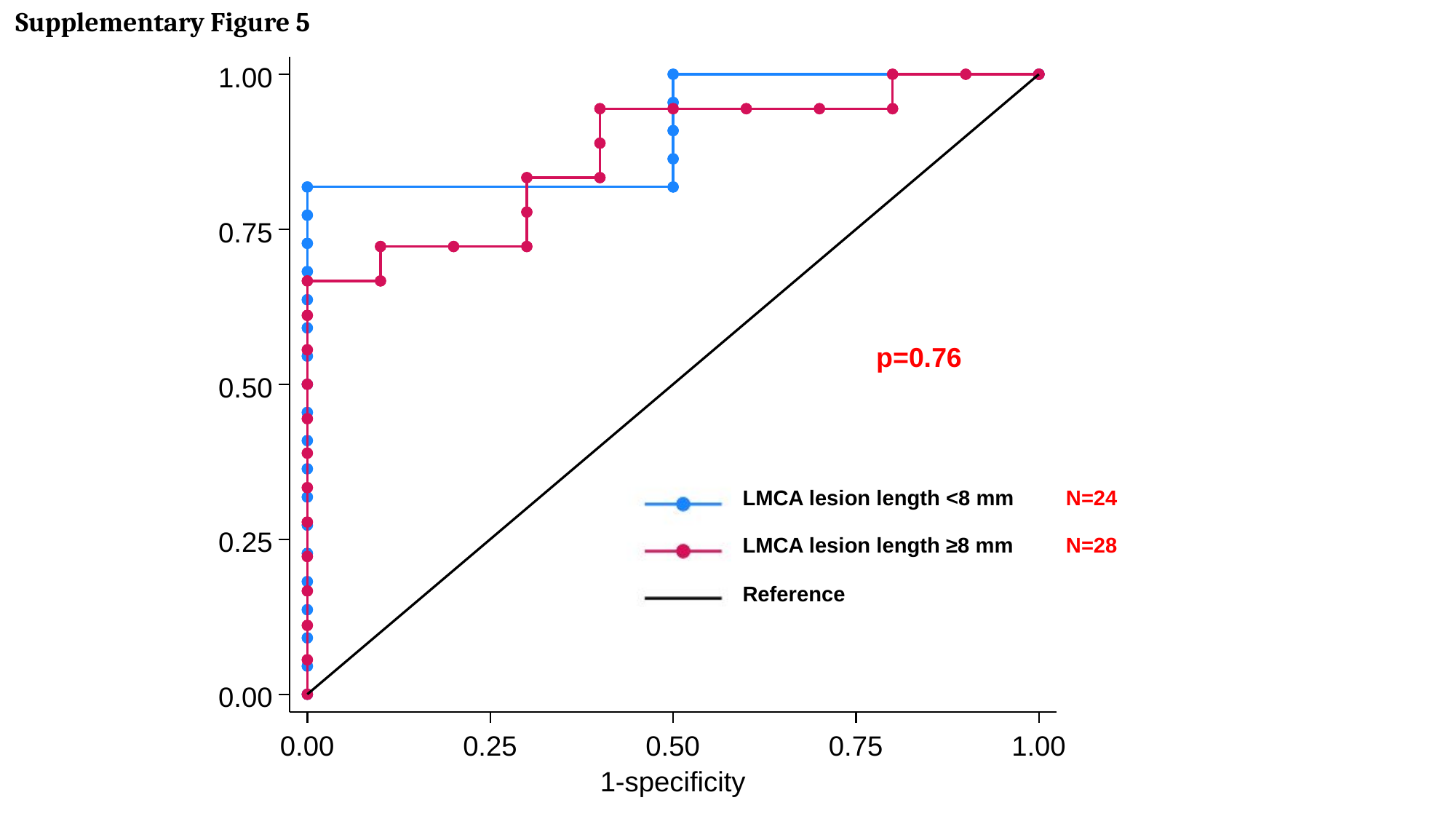

Supplementary Figure 5
p=0.76
| LMCA lesion length <8 mm | N=24 |
| --- | --- |
| LMCA lesion length ≥8 mm | N=28 |
| Reference | |

Supplement: Supplementary file 5 — Supplementary Figure 5. [file CCD-106-2207-s002.pptx]
