# Supplementary material for: Minimum Lumen Area Indexed to Left Ventricular Mass to Identify Functionally Significant Left Main Coronary Stenoses
Source: Catheter Cardiovasc Interv. 2025 Jul 30;106(4):2207–17. doi: 10.1002/ccd.70026 (PMC12502031; doi:10.1002/ccd.70026)
Supplement: Supplementary file 4 — Supplementary Figure 4. [file CCD-106-2207-s005.pptx]

## Slide 1
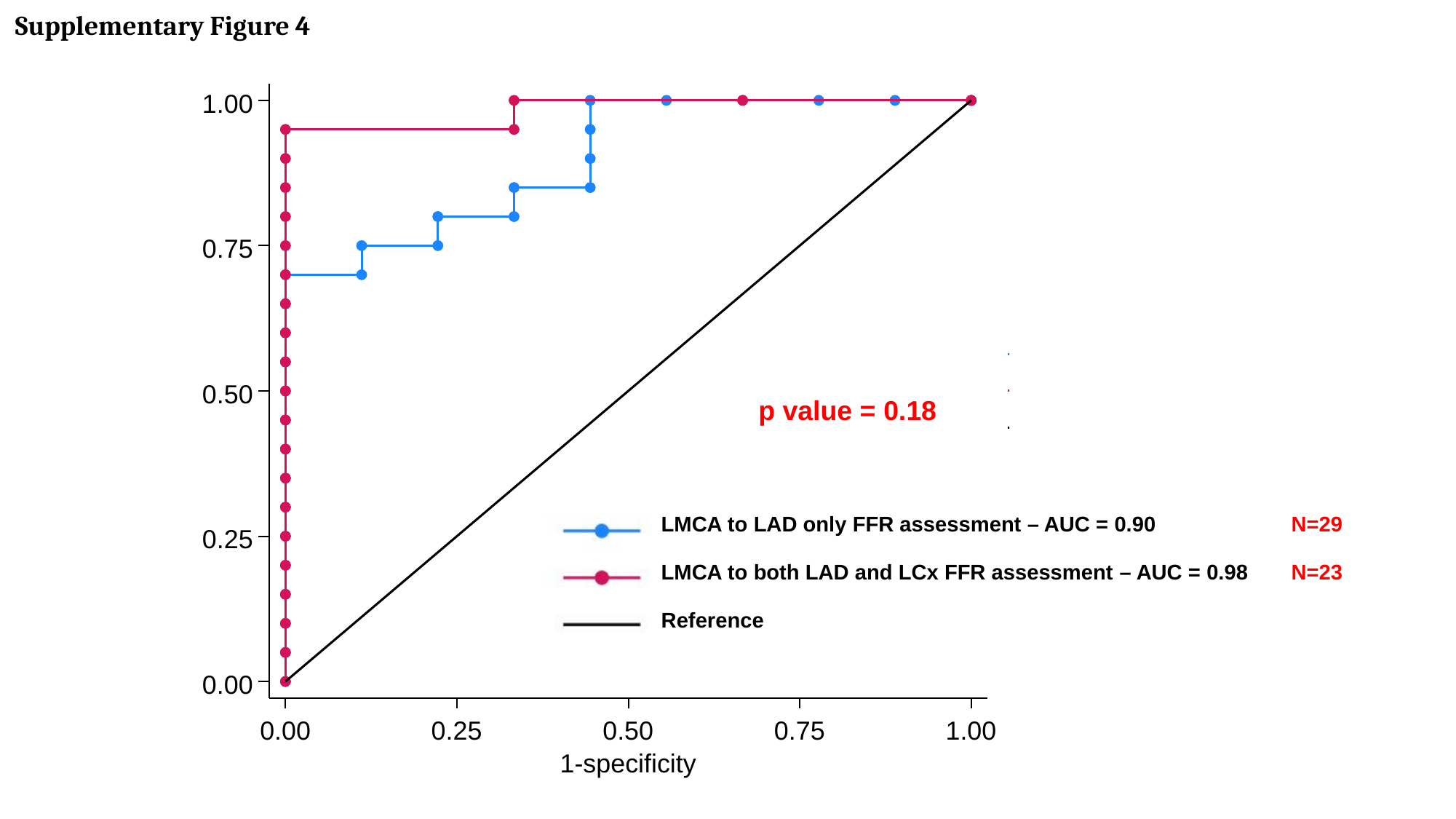

Supplementary Figure 4
p value = 0.18
| LMCA to LAD only FFR assessment – AUC = 0.90 | N=29 |
| --- | --- |
| LMCA to both LAD and LCx FFR assessment – AUC = 0.98 | N=23 |
| Reference | |
