# Supplementary material for: Minimum Lumen Area Indexed to Left Ventricular Mass to Identify Functionally Significant Left Main Coronary Stenoses
Source: Catheter Cardiovasc Interv. 2025 Jul 30;106(4):2207–17. doi: 10.1002/ccd.70026 (PMC12502031; doi:10.1002/ccd.70026)
Supplement: Supplementary file 6 — Supplementary Table 1: Characteristics of the study population stratified by MLA/LV mass threshold (29 mm2/kg). [file CCD-106-2207-s006.docx]

**Supplementary Table 1. Characteristics** **of the study population stratified by MLA/LV mass threshold (29 mm²/kg)**

|  | **Overall**  **(n=52)** | **MLA/LV mass ≥29**  **(n=27)** | **MLA/LV mass <29**  **(n=25)** | **p value** |
| --- | --- | --- | --- | --- |
| ***Demographic and anthropometric characteristics*** | |  |  |  |
| Age (years) | 69 [65–77.5] | 69 [63–79] | 70 [67–77] | 0.67 |
| Male | 40 (76.9) | 19 (70.4) | 21 (84.0) | 0.24 |
| Height (cm) | 169.87±8.45 | 169.19±9.56 | 170.60±7.19 | 0.55 |
| Weight (kg) | 75.79±14.42 | 73.81±14.30 | 77.92±14.53 | 0.31 |
| BMI (kg/m²) | 25.41 [23.77–28.52] | 24.91 [22.84–29.00] | 25.47 [24.49–27.34] | 0.42 |
| BSA (m²) | 1.88±0.21 | 1.85±0.22 | 1.92±0.20 | 0.28 |
| ***Cardiovascular risk factors and comorbidities*** |  |  |  |  |
| Systemic hypertension | 36 (69.2) | 17 (63.0) | 19 (76.0) | 0.31 |
| Dyslipidemia | 34 (65.4) | 18 (66.7) | 16 (64.0) | 0.84 |
| Diabetes | 12 (23.1) | 6 (22.2) | 6 (24.0) | 0.88 |
| Smoking | 30 (57.7) | 14 (51.9) | 16 (64.0) | 0.38 |
| CKD | 7 (13.5) | 4 (14.8) | 3 (12.0) | 0.77 |
| AF | 5 (9.6) | 3 (11.1) | 2 (8.0) | 0.70 |
| Previous PCI | 19 (36.5) | 9 (33.3) | 10 (40.0) | 0.62 |
| ***Chronic medical therapy*** |  |  |  |  |
| Beta-blockers | 39 (75.0) | 19 (70.4) | 20 (80.0) | 0.42 |
| ACEi/ARBs | 30 (57.7) | 16 (59.3) | 14 (56.0) | 0.81 |
| Nitrates | 7 (13.5) | 1 (3.7) | 6 (24.0) | **0.032** |
| CCBs | 9 (17.3) | 2 (7.4) | 7 (28.0) | **0.050** |
| Diuretics | 23 (44.2) | 11 (40.7) | 12 (48.0) | 0.60 |
| ASA | 34 (65.4) | 16 (59.3) | 18 (72.0) | 0.34 |
| P2Y12 inhibitor | 23 (44.2%) | 6 (22.2%) | 17 (68.0%) | **<0.001** |
| DOAC | 6 (11.5) | 3 (11.1) | 3 (12.0) | 0.92 |
| Statin | 30 (57.7) | 13 (48.1) | 17 (68.0) | 0.15 |
| Ezetimibe | 15 (28.8) | 8 (29.6) | 7 (28.0) | 0.90 |
| ***Echocardiographic parameters*** |  |  |  |  |
| LVEF (%) | 56.0 [49.0–61.0] | 56.0 [49.0–62.0] | 55.0 [52.0–60.0] | 0.76 |
| IVS thickness (mm) | 11.31±1.86 | 10.81±1.90 | 11.84±1.70 | **0.046** |
| PW thickness (mm) | 9.83±1.91 | 9.22±1.91 | 10.48±1.71 | **0.016** |
| EDD (mm) | 48.0 [44.0–53.5] | 47.0 [42.0–52.0] | 50.0 [45.0–55.0] | 0.11 |
| EDS (mm) | 34.30±8.24 | 33.10±8.28 | 35.45±8.23 | 0.35 |
| EDV (mL) | 98.0 [86.0–135.0] | 94.0 [75.0–130.0] | 104.0 [92.0–140.0] | 0.10 |
| ESV (mL) | 43.0 [34.0–64.0] | 40.0 [32.0–65.0] | 44.0 [35.0–61.0] | 0.38 |
| LV mass (g) | 187.42 [153.13–239.93] | 179.35 [132.10–220.00] | 224.55 [181.98–259.60] | **0.003** |
| LA volume (mL) | 55.71 [40.0–74.0] | 53.62 [40.00–80.03] | 59.84 [39.00–72.00] | 0.79 |
| TAPSE (mm) | 22.60±4.71 | 22.56±3.95 | 22.64±5.50 | 0.95 |
| ***Laboratory parameters*** |  |  |  |  |
| Hb (g/dL) | 13.91±1.78 | 13.63±1.73 | 14.21±1.82 | 0.25 |
| Serum creatinine (mg/dL) | 0.96 [0.77–1.19] | 0.96 [0.77–1.28] | 0.95 [0.75–1.19] | 0.33 |
| eGFR (mL/min/1.73 m²) | 70 [51–90] | 63.50 [51–85] | 78.00 [52–93] | 0.35 |
| LDL (mg/dL) | 86.12±36.52 | 90.48±39.43 | 82.12±33.94 | 0.43 |

Data are expressed as absolute numbers (percentages) for categorical variables and mean ± standard deviation for continuous variables with normal distribution or median [interquartile range] for continuous variables with non-normal distribution. ACEi/ARBs=Angiotensin-converting enzyme inhibitors/Angiotensin receptor blockers; ACS=Acute coronary syndrome; AF=Atrial fibrillation; ASA=Acetylsalicylic Acid; BMI=Body Mass Index; BSA=Body Surface Area; CCBs =Calcium channel blockers; CKD=Chronic kidney disease; DOAC=Direct oral anticoagulant; EDD=End-diastolic diameter; EDS=End-systolic diameter; EDV=End-diastolic volume; ESV=End-systolic volume; eGFR=estimated Glomerular filtration rate; Hb=Hemoglobin; IVS=Interventricular septum; LA=Left atrial; LDL=Low-Density Lipoprotein; LV=Left ventricular; LVEF=Left ventricular ejection fraction; MLA=Minimal lumen area; PCI=Percutaneous coronary intervention; PCSK9=Proprotein Convertase Subtilisin/Kexin Type 9; PW=Posterior wall; TAPSE=Tricuspid annular plane systolic excursion.
